# Increased binding of anti-dsDNA antibodies to short oligonucleotides modified with topoisomerase I reveals a potential new enzyme function independent from DNA relaxation

**DOI:** 10.1186/s13104-023-06592-9

**Published:** 2023-10-28

**Authors:** Manuela Frese-Schaper, Reinhard E. Voll, Steffen Frese

**Affiliations:** 1 Deutsches Rheuma-Forschungszentrum Berlin (DRFZ), a Leibniz Institute, Berlin, Germany; 2 Department of Rheumatology and Clinical Immunology, Faculty of Medicine, Medical Center-University of Freiburg, University of Freiburg, Freiburg, Germany; 3 Department of Thoracic Surgery, AMEOS Klinikum Schönebeck, Köthener Str. 13, D-39218 Schönebeck, Germany

**Keywords:** Autoimmune disease, Systemic lupus erythematosus, Topoisomerase I, DNA relaxation, Anti-dsDNA antibodies, Anti-dsDNA binding

## Abstract

**Objective:**

Topoisomerase I (topo I) is a highly conserved enzyme which is known to reduce torsional stress at double-stranded (ds) DNA. Torsional stress induced by supercoiling of dsDNA requires either very long dsDNA existing in genomic DNA or circulation as presented in plasmid DNA. To enable DNA relaxation, topo I induce a transient single-strand break followed by stress-relieving rotation of the released DNA strand. Our group found by serendipity that the topo I inhibitor irinotecan is able to suppress murine systemic lupus erythematosus (SLE), an autoimmune disease which is characterized by the existence of pathogenic anti-dsDNA antibodies (abs). As a possible mechanism we demonstrated in the absence of immunosuppression an increased binding of anti-dsDNA abs to long genomic or circulated plasmid dsDNA modified with topo I.

**Results:**

Here we show that this effect requires active site tyrosine of topo I which is known to facilitate DNA relaxation activity. Moreover, topo I enhanced anti-dsDNA abs binding to short linear oligonucleotides down to a size of 42 bp. Since oligonucleotides of such length are devoid of torsional stress and relaxation respectively, our results suggest a new and unknown function for the enzyme topo I.

**Supplementary Information:**

The online version contains supplementary material available at 10.1186/s13104-023-06592-9.

## Introduction

Topo I belongs to the type IB subfamily of topoisomerases. The enzyme is expressed in all eukaryotic cells and is essential for the development of higher eukaryotic organisms since homozygous deletion mutants were shown to be lethal at an early embryonic stage [1, 2]. Topo I is known to reduce torsional stress that develops during replication of DNA. To achieve this, topo I binds to both negatively and positively supercoiled duplex DNA and induces transient single-stranded (ss) DNA breaks. Strand cleavage is carried out by formation of a phosphodiester bond between the active site tyrosine of topo I (in human at position 723) and the 3´end of the cleaved strand. Cleavage is followed by stress-relieving rotation of the nicked DNA strand (DNA relaxation). Then, ssDNA breaks are re-ligated by topo I to reconstitute intact dsDNA [3–5]. Interestingly, re-ligation of dsDNA seems to be independent from the active site tyrosine at position 723 [6]. In addition to DNA relaxation activity, a protein kinase activity in the context with RNA splicing has been described for topo I. Thus, topo I specifically phosphorylated serine-arginine rich splicing factors thereby changing their activity [7]. Remarkably, the kinase activity of topo I was inhibited by the topo I inhibitor camptothecin in the presence of DNA but was independent of the active site tyrosine at position 723 needed for DNA relaxation activity [8].

More than a decade ago our group found by serendipity that the topo I inhibitor irinotecan suppressed the autoimmune disease SLE in mice [9]. Inhibitors of topo I such as irinotecan or rather its active component SN-38 are known as chemotherapeutic agents for many years. They bind to the complex of topo I and DNA (and not to DNA or to topo I alone) named the cleavable complex [10]. Binding of the topo I inhibitor stabilizes the cleavable complex and prevents re-ligation of DNA. In dividing cells with enhanced DNA replication the complex of DNA, topo I and its inhibitor can collide with DNA replication forks existing only during S-phase. Collision of the cleavable complex with DNA replication forks results in the generation of irreversible dsDNA breaks followed by the induction of cell death [11, 12]. By this mechanism inhibitors of topo I induce cell death (apoptosis) in proliferating cancer cells; therefore the topo I inhibitor irinotecan is used for the treatment of metastatic cancer [13, 14].

After the initial finding of irinotecan on murine lupus, subsequent experiments of our group demonstrated that the dosages needed to reverse advanced lupus nephritis in mice were more than 50 times lower than the doses required for chemotherapy [15]. In line with this observation, we demonstrated that treatment of lupus-prone mice with irinotecan did not induce profound immunosuppression [9, 15–17], in contrast to strong immunosuppressive treatments which are normally used to treat severe SLE flares such as lupus nephritis [18–23]. Since immunosuppression was ruled out, we looked for other mechanisms to answer the question how the topo I inhibitor irinotecan suppressed an autoimmune disease which exhibits anti-dsDNA abs as the main pathogen. In vitro studies indicated that topo I changed binding of anti-dsDNA abs. For these experiments we used either very long DNA such as calf thymus (ct) and nucleosomal DNA assuming a supercoiled state, or we utilized supercoiled plasmid DNA having a ring structure. When treating these different preparations of DNA with recombinant topo I, binding of anti-dsDNA abs was markedly increased [15–17]. This effect was inhibited by the topo I inhibitor camptothecin [15, 16]. We concluded from these results that topo I-mediated relaxation of dsDNA enhanced binding of anti-dsDNA abs. However, some first doubt came up when using poly(dA-dT) DNA for these experiments. Unexpectedly, topo I also increased binding of anti-dsDNA abs to poly(dA-dT) DNA of less than 1 kb length, which should not form a supercoiled structure [15]. Consequently we asked ourselves whether indeed DNA relaxation or possibly other, so far unknown processes were involved in enhanced binding of anti-dsDNA abs to dsDNA modified by topo I.

## Materials and methods

### Antibodies, DNA and topo I

Monoclonal anti-dsDNA abs (clones 3C5, 8F4, 9A10, 8H4 and 1E5) were propagated, harvested and purified as described before [24]. The anti-dsDNA ab HYB331-01 was provided by the Statens Serum Institut, Denmark. Fab fragments of HYB331-01 were generated by papain digestion followed by purification using protein A–Sepharose affinity chromatography [25, 26]. ctDNA was obtained from Sigma-Aldrich, supercoiled plasmid DNA pBR322 was from Inspiralis. Oligonucleotides were purchased from biomers net GmbH. Active human topo I (UniProtKB P11387) was provided by ProSpec, catalytically inactive human topo I (mt.Y723F) was from TopoGEN.

### Effect of DNA modification on anti-dsDNA-IgG binding

ctDNA was passed through an Millex-HA 0.45 mm filter (Millipore) to eliminate ssDNA. Indicated amounts of ctDNA or supercoiled plasmid DNA were incubated with recombinant human topo I in 40 mM Tris-HCl, pH 7.5, 100 mM KCl, 10 mM MgCl_2_, 0.5 mM DTT, 0.5 mM EDTA, 30 µg/ml BSA for 30 min at 37 °C. Then, 30 µl of sample per well was used for coating a 384-well Nunc MaxiSorp plates overnight at 4 °C. Plates were blocked with PBS containing 1% casein (Pierce) for 1 h at 37 °C, and plates were incubated with monoclonal anti-dsDNA abs at a concentration of 1 µg/ml for 1 h at 37 °C. Subsequently, plates were incubated with alkaline phosphatase-conjugated goat anti-mouse IgG (Southern Biotech) for 1 h at 37 °C and developed with p-nitrophenol phosphate (Sigma-Aldrich). Optical density (OD) was measured at 405 nm with a reference filter at 490 nm. When used oligonucleotides for these experiments, forward and reverse strands were annealed for 3 min at 93 °C. After incubation with recombinant topo I as described above, modified oligonucleotides were attached to Nunc MaxiSorp plates overnight at 4 °C using DNA Coating Solution from Pierce. Detection of anti-dsDNA binding was performed as described above.

### Extent of DNA relaxation

To visualize the extent of DNA relaxation, modified plasmid DNA samples were run on 1% agarose gel and visualized with GelRed™ (Biotium).

### Statistical analyses

Data were analyzed using either one-way analysis of variance with Bonferroni’s post hoc test or unpaired t-test with Welch’s correction. Some data were assessed with Mann-Whitney test. For all tests the software GraphPadPrism version 9.4 was used. Differences were considered statistically significant at P values < 0.05.

## Results

**Fig. 1:**
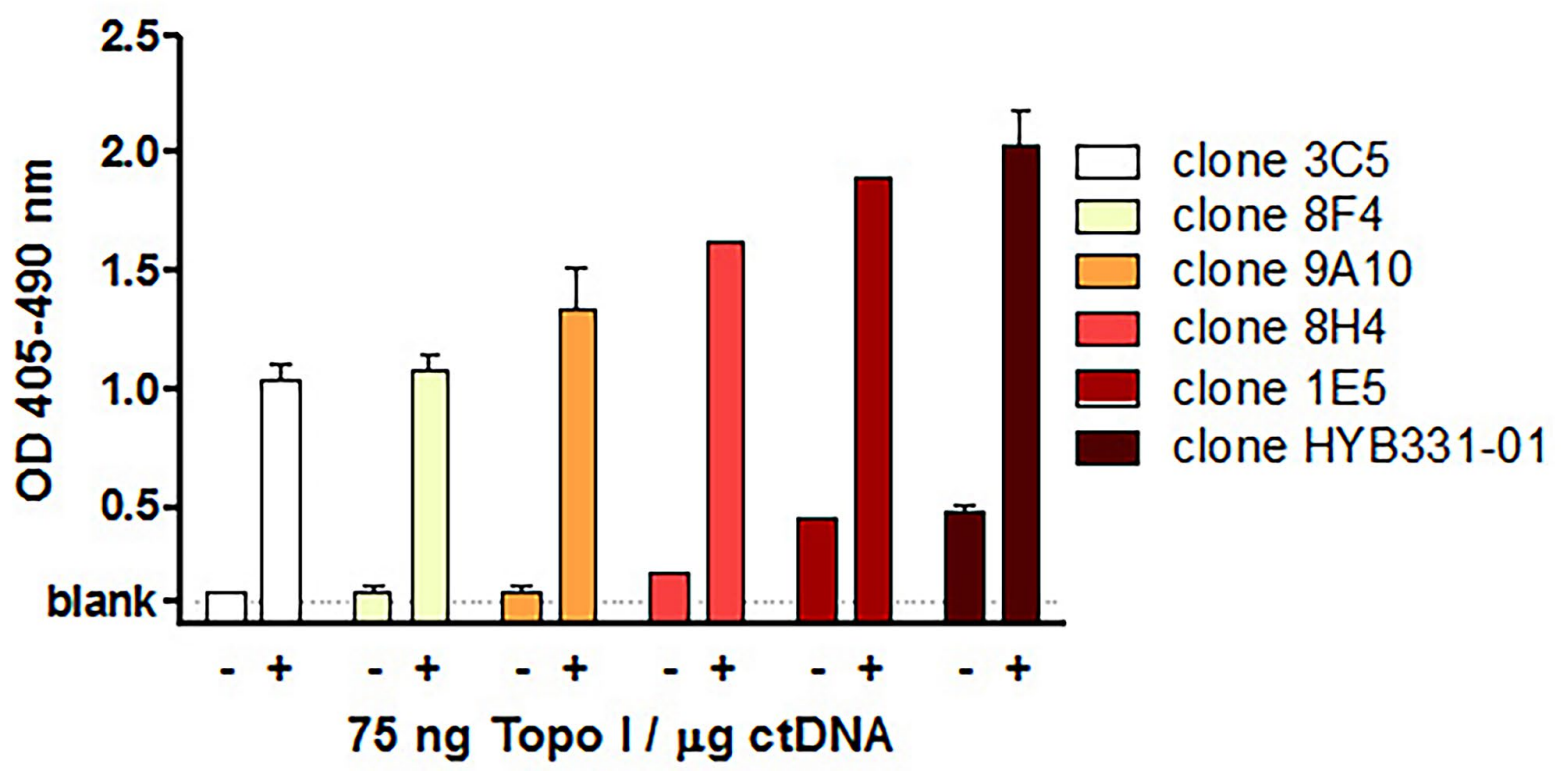
Increased binding of different clones of anti-dsDNA abs to ctDNA modified with recombinant topo I. Modification of ctDNA was performed using 75 ng topo I per µg DNA. 384-well plates were than coated with 1.5 µg of modified DNA and incubated with 30 ng of anti-dsDNA abs. The extent of anti-dsDNA binding was measured by ELISA. Bars express the mean ± SD of at least two independent experiment performed in duplicates. All probes modified with topo I showed a significant higher anti-dsDNA abs binding using the Mann-Whitney test for statistical analysis

Our previous work demonstrated enhanced binding of the monoclonal anti-dsDNA ab HYB331-01 to ctDNA modified with recombinant topo I. The same effect was shown for anti-dsDNA abs from mice and humans suffering from SLE [15]. In order to uncover the underlying mechanism we tested a panel of other monoclonal anti-dsDNA abs. As shown in Fig. 1 all anti-dsDNA abs showed significantly enhanced binding to ctDNA which was modified by topo I.

**Fig. 2:**
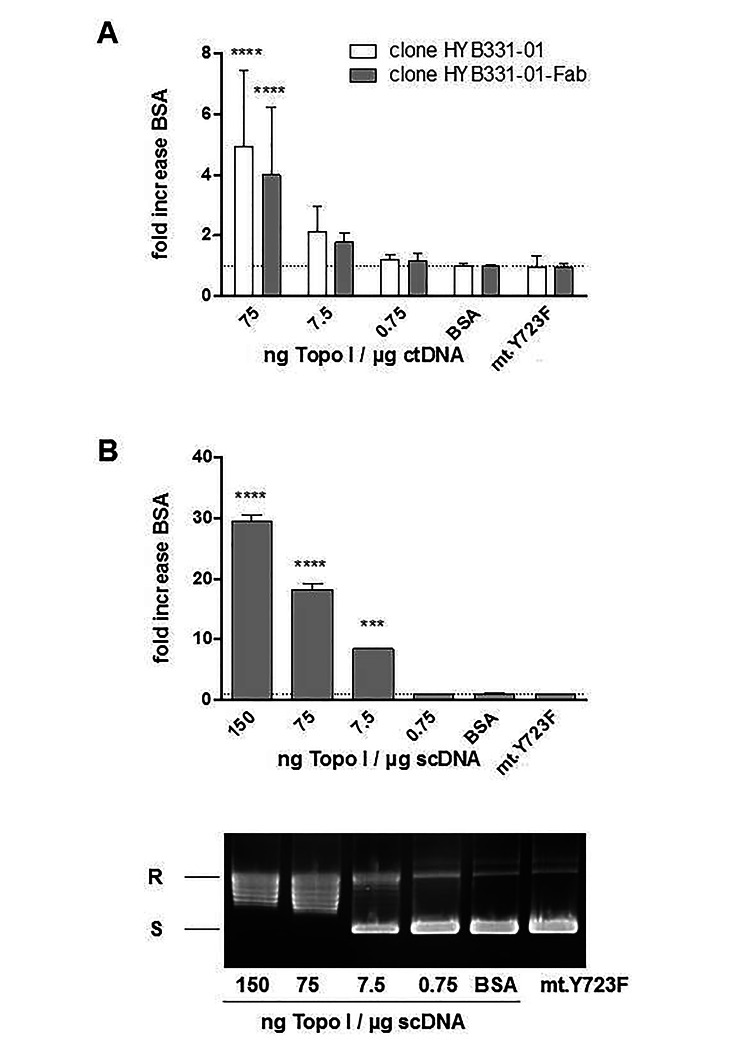
Increased binding of anti-dsDNA ab HYB331-01 full length or as Fab fragment to ctDNA or supercoiled plasmid DNA modified with catalytically active topo I. Modification of ctDNA or supercoiled DNA was performed using the indicated amount of topo I per µg DNA. Mutated topo I Y723F was used with 75 ng per µg DNA. 384-well plates were than coated with either 12.5 µg of modified ctDNA (**A**) or 4 µg of modified supercoiled plasmid DNA followed by incubation with 30 ng of anti-dsDNA ab HHB331-01 full length (**A**) or as Fab fragment (**B**). The extent of anti-dsDNA binding was measured by ELISA. Bars express the mean ± SD of at least two independent experiment performed in quadruplicates (**A**) or duplicates (**B**). One-way ANOVA with Bonferroni’s post hoc test. ***P<0.001, ****P<0.0001

Next we asked whether Fab fragments of anti-dsDNA abs behave similar to full length antibodies since previous publication suggested a decreased binding by monogamous bivalency [27]. We also looked whether this effect requires topo I activity. As the result we were able to show that binding of the Fab fragment of HYB331-01 anti-dsDNA ab to ctDNA was enhanced when DNA was modified with topo I. The effect was dependent from the amount of topo I and diminished when lowering topo I concentrations (Fig. 2A). Same effects were seen when using supercoiled plasmid DNA (Fig. 2B). When DNA was modified with topo I mutated at the active site tyrosine (Y723F), no effect was observed on anti-dsDNA abs binding. (Fig. 2A;B). Since it is known that inactive mutant topo I Y723F is not able to cleave and relax DNA [6], the data strongly suggest that DNA cleavage is necessary for topo I-mediated enhancement of anti-dsDNA abs binding.

To answer the question whether DNA relaxation is responsible for increased binding of anti-dsDNA abs, we investigated the effect on ds oligonucleotides of short length. The rationale for this approach was that oligonucleotides of short length are devoid of supercoiling and DNA relaxation respectively.

**Fig. 3:**
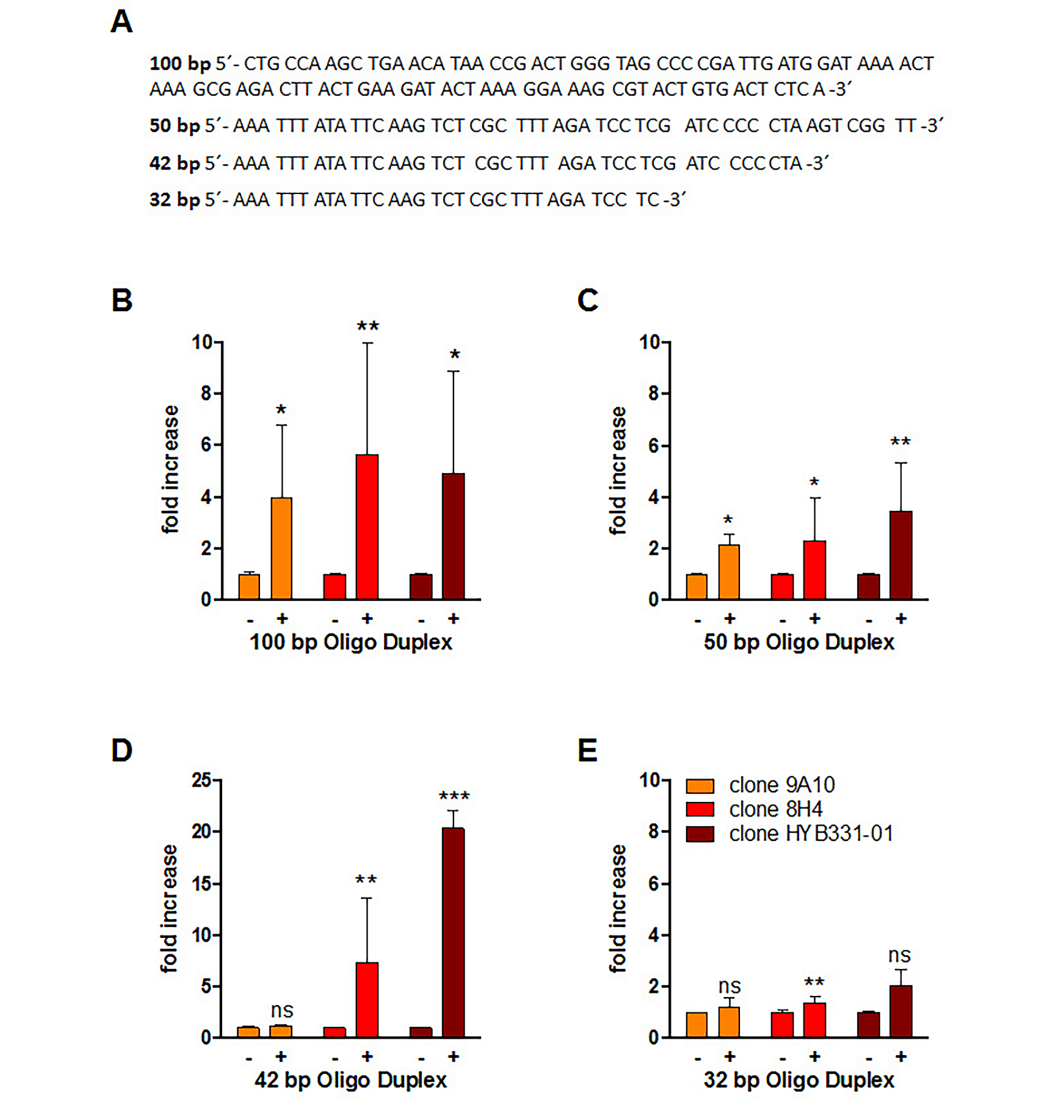
Increased binding of of anti-dsDNA abs to short oligonucleotides modified with recombinant topo I. (**A**) Sequence of oligonucleotides used for the expreiments. (**B**)-(**E**) Modification of oligonucleotides was performed using 100 ng topo I per µg DNA. 384-well plates were than coated with 10 or 20 µg of modified DNA and incubated with 30 ng of anti-dsDNA abs. The extent of anti-dsDNA binding was measured by ELISA. Bars express the mean ± SD of at least two independent experiment performed in duplicates. Unpaired t test with Welch’s correction was used for statistical analysis. *P<0.05, **P<0.01, ***P<0.001

As Fig. 3 demonstrates, treatment of oligonucleotides down to a length of 42 bp with topo I increased significantly anti-dsDNA abs binding. The effect decreased clearly when using a 32 bp oligonucleotide. Whether the diminishing effect was real or whether it was a problem of detection cannot be definitely answered thus far. However, the data strongly suggest that albeit strand cleavage is necessary for topo I-mediated enhancement of anti-dsDNA abs binding, DNA relaxation is not involved in this process.

## Discussion

The function of topo I is far away from being fully understood. This somewhat provoking statement might be explained by conflicting data generated more than two decades ago demonstrating an additional topo I kinase activity. Topo I was shown to specifically phosphorylate serine-arginine rich splicing factors and this effect was inhibited by the topo I inhibitor camptothecin but only in the presence of DNA [7]. However, using topo I mutated at the active site tyrosine (Y723F) which is unable to cleave DNA and to promote DNA relaxation, the topo I mutant was still able to phosphorylate serine-arginine rich splicing factors [8].

A new part to the topo I enigma was added when our group found by serendipity that the topo I inhibitor suppressed the autoimmune disease SLE in mice. SLE is characterized by the presence of autoantibodies directed against dsDNA [28] affecting mainly women of childbearing age [29]. Immunosuppression, either by general immunosuppression or by depletion of specific immune cells such as B cells has been applied for many years to treat SLE [21, 22, 30]. With respect to the extremely low doses used for our animal experiments we demonstrated that immunosuppression was not involved in topo I mediated suppression of murine SLE [9, 15]. To look for mechanisms different from immunosuppression we found out that topo I and its inhibitor changed binding of anti-dsDNA abs. These mechanistic data were published in 2014 [15]. However, despite suppression of SLE in mice by inhibition of topo I has been approved by an independent group [31], first patients with refractory lupus have been treated with irinotecan successfully [32, 33] and also, while topo I inhibitors have been suggested by computational drug repurposing as a target for the treatment of SLE recently [34, 35], the demonstrated effects of topo I on anti-dsDNA abs binding have not drawn interest to the scientific or clinical community yet.

While we thought in the beginning that topo I-mediated effects on anti-dsDNA binding is a consequence of altered DNA relaxation [15], the effects demonstrated here contradict this hypothesis. Since short oligonucleotides cannot undergo supercoiling and torsional stress, we postulate a new function for topo I independent of DNA relaxation. The new function of topo I requires most likely topo I cleavage acitivity as shown by mutant topo I (Y723F) but is not related to DNA relaxation which is believed to be a consequence of topo I-mediated DNA cleavage. Furthermore, phosphorylation activity of topo I can be ruled out as well to be involved. Whereas topo I mutated at the active site tyrosine was still able to facilitate phosphorylation activity, mutant topo I did not increase anti-dsDNA abs binding. More experiments are needed do uncover the exact mechanism of the predicted new function of topo I.

### Limitations

Increased binding of anti-dsDNA abs to short dsDNA was shown just by ELISA and no other methods. The conclusion that DNA relaxation can be ruled out as mechanism for topo I-mediated changes in anti-dsDNA binding is indirect and the proposed new function of topo I cannot be specified further. To get answered these questions our group tried to generate crystals of anti-ds abs and DNA modified with topo I but did not succeed thus far.

### Electronic supplementary material

Below is the link to the electronic supplementary material.

Supplementary Material 1

## Data Availability

The datasets used and/or analyzed during the current study are available from the corresponding author on request.

## Abbreviations

topo I: topoisomerase I
SLE: systemic lupus erythematosus
ds: double-stranded
abs: antibodies
bp: base pair
ct: calf thymus
sc: supercoiled
