# Supplementary figures and images for: Increased binding of anti-dsDNA antibodies to short oligonucleotides modified with topoisomerase I reveals a potential new enzyme function independent from DNA relaxation
Source: BMC Res Notes. 2023 Oct 28;16:298. doi: 10.1186/s13104-023-06592-9 (PMC10612351; doi:10.1186/s13104-023-06592-9)

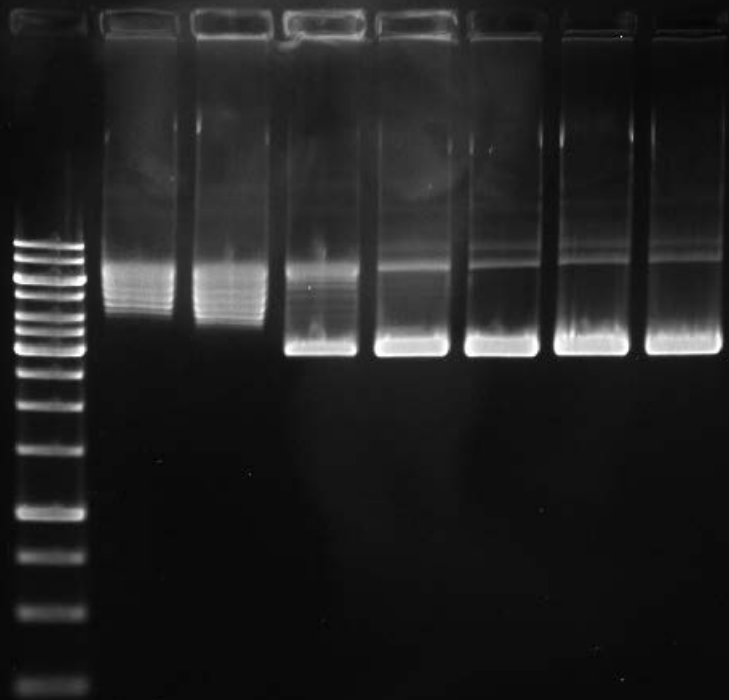

Supplement: Supplementary file 1 — Supplementary Material 1 [file 13104_2023_6592_MOESM1_ESM.pdf]
